# Chrysin Mitigates Acetamiprid‐Induced Testicular Injury in Mice via Suppression of Endoplasmic Reticulum Stress and Inflammation

**DOI:** 10.1002/tox.70076

**Published:** 2026-03-16

**Authors:** Selim Demir, Nihal Turkmen Alemdar, Esin Yulug, Ali Kulaber, Elif Ayazoglu Demir, Nadire Sevdenur Erdogan, Ahmet Mentese, Yuksel Aliyazicioglu

**Affiliations:** ^1^ Department of Medical Biochemistry, Faculty of Medicine Karadeniz Technical University Trabzon Turkiye; ^2^ Department of Medical Services and Techniques Vocational School of Health Services, Recep Tayyip Erdogan University Rize Turkiye; ^3^ Department of Histology and Embryology, Faculty of Medicine Karadeniz Technical University Trabzon Turkiye; ^4^ Department of Chemistry and Chemical Processing Technologies Macka Vocational School, Karadeniz Technical University Trabzon Turkiye

**Keywords:** acetamiprid, chrysin, endoplasmic reticulum stress, inflammation, Nrf2, oxidative stress, testicular toxicity

## Abstract

Acetamiprid (ACP) is a neonicotinoid pesticide that is extensively utilized for the management of insect populations, but its toxic effects on the male reproductive system have become a source of concern. Chrysin (CHR) is a natural flavone found in many plants and bee products, including honey and propolis. The objective of this study was to evaluate the protective effect of CHR on ACP‐induced testicular injury in male Balb/c mice. ACP (20 mg/kg) and CHR applications (10 and 20 mg/kg, respectively) were conducted via intragastric route for a period of 14 days. The administration of CHR treatments resulted in a substantial improvement in oxidative stress and inflammation levels, achieved by the elimination of ACP's inhibition of the Nrf2 pathway. CHR treatments significantly reversed the ACP‐induced depletion in serum testosterone and inhibin B levels. Specifically, the treatment of CHR at a dose of 20 mg/kg resulted in a substantial improvement in degenerative testicular findings induced by ACP, as indicated by a significant reduction in TUNEL (+) cell count. Furthermore, the provision of CHR treatments resulted in the cessation of the decline in sperm count and viability that had been induced by ACP, whilst concomitantly leading to a substantial reduction in the number of spermatozoa that exhibited severe abnormalities. These results suggest that CHR may have a protective effect against ACP‐induced testicular damage; however, the results require further corroboration through the implementation of more comprehensive studies.

**Abbreviations**
ACP: acetamiprid
AI: apoptotic index
ATF6: activating transcription factor 6
cAMP: cyclic adenosine monophosphate
CASP3: cleaved caspase‐3
CHR: chrysin
CMC: carboxymethylcellulose
DDIT3: DNA damage‐inducible transcript 3
DMSO: dimethyl sulfoxide
ELISA: enzyme‐linked immunosorbent assay
ERS: endoplasmic reticulum stress
GCLC: glutamate–cysteine ligase catalytic subunit
GPx: glutathione peroxidase
GSH: glutathione
H&E: hematoxylin and eosin
HMGB1: high mobility group box 1
HO‐1: heme oxygenase‐1
HSPA5: heat shock protein family A member 5
IL‐6: interleukin‐6
INHB: inhibin B
LPO: lipid peroxidation
MDA: malondialdehyde
MPO: myeloperoxidase
nAChRs: nicotinic acetylcholine receptors
NF‐κB: nuclear factor kappa‐B
Nrf2: nuclear factor erythroid 2‐related factor 2
OS: oxidative stress
PAS: periodic acid Schiff staining
ROS: reactive oxygen species
SEM: standard error of the mean
SOD: superoxide dismutase
TES: testosterone
TUNEL: terminal deoxynucleotidyl transferase dUTP nick end labeling
UPR: unfolded protein response

## 1 Introduction

Reproduction is one of the physiological processes in the organism that is most sensitive to adverse environmental conditions, and pesticides have become a significant concern today because they cause problems with reproduction [1]. Acetamiprid (ACP) is a neonicotinoid pesticide that is employed to protect agricultural products and ornamental plants from insect infestation. It was first synthesized in 1984 and approved for use in 2002 [2]. The insecticidal properties of ACP are attributable to its capacity to function as an agonist of nicotinic acetylcholine receptors (nAChRs) [1]. This results in the disruption of neurotransmission and alteration of membrane potential, ultimately leading to the paralysis and death of the affected organism [3]. The studies have demonstrated that ACP exhibits a substantial affinity for mammalian nAChRs, which is the underlying cause of its toxicity to mammalian tissues [1]. It has been demonstrated through experimental studies that exposure to ACP results in toxicity to the hematopoietic, nervous, hepatic, renal, immune, and reproductive systems in mammals [3].

The reproductive system is one of the tissues in which nAChRs are most extensively distributed; as a result, it is particularly susceptible to detrimental effects caused by ACP toxicity [1]. It has been determined that ACP exposure leads to the occurrence of histopathological findings in experimental animals, as well as a reduction in serum testosterone (TES) levels and sperm count [3]. While oxidative stress (OS) resulting from the suppression of the antioxidant system and the increase in reactive oxygen species (ROS) plays a central role in the biochemical mechanism of ACP toxicity, other mechanisms, such as inflammation, endoplasmic reticulum stress (ERS), and apoptosis, also contribute to the formation of tissue damage [1, 3, 4]. To protect themselves from the damaging effects of OS, cells have developed enzymatic defense systems; consisting mainly of superoxide dismutase (SOD), catalase (CAT), and glutathione peroxidase (GPx), and non‐enzymatic defense systems; consisting mainly of glutathione (GSH) [5]. Experimental studies have shown that ACP increases lipid peroxidation (LPO) and ROS formation, reduces mitochondrial membrane potential, depletes the GSH pool, and inhibits the activity of SOD and GPx enzymes [3]. It is also well documented that ACP‐induced ROS increase activates the high mobility group box 1 (HMGB1)/nuclear factor kappa‐B (NF‐κB) axis and increases pro‐inflammatory cytokines such as interleukin‐6 (IL‐6), which in turn causes persistent inflammatory processes [6, 7, 8]. The ER is a very important organelle for protein synthesis, folding, and transport [9]. The disruption of ER protein folding potential as a result of increased OS and inflammation is known as ERS [10]. There is increasing evidence that ACP can activate apoptotic processes by increasing ERS [4, 11]. One of the principal regulators of antioxidant defense mechanisms is nuclear factor erythroid 2‐related factor 2 (Nrf2) [12]. Increased ROS levels have been shown to facilitate the dissociation of Nrf2 from Kelch‐like ECH‐associated protein 1 (Keap1), a regulatory protein [13]. The released Nrf2 then translocates to the nucleus, where it increases the transcription of numerous antioxidant and anti‐inflammatory genes, including heme oxygenase‐1 (HO‐1), SOD, GPx, and glutamate‐cysteine ligase catalytic subunit (GCLC) [12]. There is increasing evidence that ACP reduces tissue resistance to oxidative damage, inflammation, and apoptosis by reducing the expression of Nrf2 and its target proteins [8, 14]. Therefore, the identification of molecules capable of reducing OS, inflammation, and apoptosis by eliminating ACP‐induced Nrf2 pathway inhibition is regarded as a targeted approach to eliminate ACP toxicity [14].

Natural products constitute a significant traditional source of nutrition and medicine, with a considerable proportion of contemporary pharmaceuticals being derived from natural sources [15]. Chrysin (CHR) is a flavone that occurs naturally in a variety of plant, fungal, and bee products, including honey and propolis [16]. It has been well documented that it has antidiabetic, antioxidant, antiapoptotic, anticancer, and anti‐inflammatory activities [15]. CHR has been demonstrated to exhibit tissue and organ protective effects by modulating the Nrf2 pathway, especially in pathological conditions accompanied by OS and inflammation [16]. Furthermore, CHR has been demonstrated to exert a protective effect on the testicles in models of testicular injury induced by methotrexate [17], cadmium [18], and cyclophosphamide [19]. However, there is a paucity of research demonstrating the potential effects of CHR on testicular ACP toxicity. On the basis of the above, the hypothesis was formulated that CHR can prevent ACP‐induced testicular damage; and this hypothesis was subsequently tested using biochemical and histopathological methods in an experimental mouse model in this study.

## 2 Materials and Methods

### 2.1 Chemicals and Reagents

The ACP (98% purity) was provided by Hektaş Ticaret T.A.Ş. (Istanbul, Turkey) and freshly suspended in a solution of 0.5% carboxymethylcellulose (CMC) on each study day [20, 21]. The CHR was obtained from Sigma–Aldrich (St. Louis, MO, USA) and dissolved in a solution comprising 10% dimethyl sulfoxide (DMSO) and 0.5% CMC. This solution was prepared on a daily basis [22, 23]. The enzyme‐linked immunosorbent assay (ELISA) kits used for the quantification of biochemical parameters were obtained from Fine Biotech (Wuhan, China). The terminal deoxynucleotidyl transferase dUTP nick end labeling (TUNEL) assay kit was purchased from (Roche, Mannheim, Germany). All other chemicals were analytical grade and purchased from Sigma–Aldrich (St. Louis, MO, USA).

### 2.2 Animals and Experimental Protocol

This experimental study was conducted in accordance with the ARRIVE guidelines and the UK Animals Act 1986, and all experimental procedures were approved by the Animal Experiments Local Ethics Committee of Karadeniz Technical University (approval no: 2024/17). Thirty male BALB/c mice (20–22 g body weight) were sourced from the Surgical Application Research Center of Karadeniz Technical University, and all experimental procedures were conducted at this facility. The mice were housed under standard laboratory conditions, which included a temperature of 25°C, humidity of 60%, and a 12 h light–dark cycle. They were provided with ad libitum access to standard mouse chow and water. Following a 1 week acclimatization period, the mice were randomly assigned to one of five groups and all treatments were administered via gavage for a period of 14 days.

The *control group* was administered 0.5% CMC via gavage on a daily basis. The *high‐dose CHR group* received a daily gavage dose of 20 mg/kg of CHR. The *ACP group* received a daily gavage dose of 20 mg/kg of ACP. The dose of ACP employed in the study was determined by considering the relatively mild dose that has been demonstrated to exert toxic effects on the testicle in previous experimental studies [24, 25]. The *ACP + CHR (10 mg/kg)* group were administered ACP (20 mg/kg) once a day via gavage, and 2 h later, CHR (10 mg/kg) was administered via gavage. The *ACP + CHR (20 mg/kg)* group were administered ACP (20 mg/kg) once a day via gavage, and 2 h later, CHR (20 mg/kg) was administered via gavage. The doses of CHR used in the study were determined based on previous reports that demonstrated the antioxidant and anti‐inflammatory activity of CHR in experimental models of zearalenone‐induced testicular toxicity [26] and cerebral ischemia/reperfusion injury [27].

### 2.3 Sampling

Twenty‐four hours after the last treatments were administered, blood samples were collected via cardiac puncture under general anesthesia (a combination of ketamine and xylazine) into serum tubes with separator gel. Following clotting, the blood samples were subjected to centrifugation at 1800*g* for a period of 10 min. The serum specimens thus obtained were then utilized in the subsequent hormone analysis. Then, testicular and epididymal tissues were immediately removed. Testicular tissues were utilized in biochemical and histological analyses, while epididymal tissues were employed in sperm analysis. Testicular tissues were homogenized in ice‐cold phosphate buffered saline (10% w/v), then subjected to centrifugation. The protein content of the resulting supernatants was quantified using bicinchoninic acid protein assay [28].

### 2.4 Measurement of LPO

The level of LPO in testicular tissue was quantified using the thiobarbituric acid test, as described by Mihara and Uchiyama [29]. Briefly, the supernatant was mixed with phosphoric acid and thiobarbituric acid in a glass tube and incubated at 90°C for 1 h. Then, the tubes were cooled, centrifuged, and the absorbance of the transparent supernatant was measured at 532 nm. The standard employed was 1,1,3,3‐tetramethoxypropane, with the results expressed in nmol/mg protein [30].

### 2.5 Measurement of Antioxidant Biomarkers

The levels of SOD, GSH, GPx, Nrf2, HO‐1, and GCLC in testicular tissue were determined using ELISA kits, in accordance with the methodology specified by the manufacturer.

### 2.6 Measurement of Inflammation Biomarkers

The levels of HMGB1, NF‐κB p65, IL‐6, and myeloperoxidase (MPO) in testicular tissue were determined using ELISA kits, in accordance with the methodology specified by the manufacturer.

### 2.7 Measurement of ERS and Apoptosis Biomarkers

The levels of heat shock protein family A member 5 (HSPA5), activating transcription factor 6 (ATF6), DNA damage‐inducible transcript 3 (DDIT3), and cleaved caspase‐3 (CASP3) in testicular tissue were determined using ELISA kits, in accordance with the methodology specified by the manufacturer.

### 2.8 Measurement of Serum Reproductive Hormones

Serum TES and inhibin B (INHB) levels were quantified using commercial ELISA kits, following the manufacturer's instructions and the resulting TES and INHB concentrations were expressed as ng/mL and pg/mL, respectively.

### 2.9 Histological Analysis

Testicular tissue samples were fixed in Bouin's solution for histological examination and embedded in paraffin blocks following the standard tissue processing procedure. Five micrometer‐thick sections were taken from each block [31]. The sections were subjected to hematoxylin–eosin (H&E) staining to evaluate the general histological structure and periodic acid–Schiff (PAS) staining to enhance the visibility of the seminiferous tubule basement membranes. Histological examinations were performed under a light microscope by a histologist who was unaware of the experimental groups. The slides were evaluated and photographed by averaging the analysis performed on a minimum of five images taken from each section under a light microscope at 200× and 400× magnifications. The pathological findings in testicular tissues were evaluated in terms of the criteria of frequency of germinal epithelial cells observed in the lumen, accumulation of seminiferous tubule epithelium towards the lumen, vasocongestion, irregularity, and dissociation between seminiferous tubule epithelial cells [32]. In addition, Johnsen's tubular biopsy scoring system was utilized to evaluate the structure of seminiferous tubules [33].

### 2.10 TUNEL Assay

The level of apoptosis in seminiferous tubule epithelial cells in testicular tissue was determined by TUNEL method using a commercial kit according to the manufacturer's instructions. Cells with brown‐colored nuclei were defined as TUNEL (+) apoptotic cells. Following the determination of the number of TUNEL (+) cells and the total number of cells in 20 seminiferous tubules in each testis, the apoptotic index (AI) was calculated using the following formula [34]:

$$\mathrm{AI}=\left(\text{TUNEL}\;\left(+\right)\;\text{cell number}/\text{total cell number}\right)\times 100$$

### 2.11 Epididymal Sperm Analysis

The epididymis was gently lysed in a petri dish containing 2 mL of tris buffer solution (pH: 7.5). The epididymis was then placed into an incubator set at 37°C for a period of 30 min, with the objective of enabling the sperm cells to become suspended within the liquid. Subsequent evaluation of sperm count, viability, and morphology was conducted on samples obtained from this liquid using a light microscope (Olympus BX‐51, Tokyo, Japan). The sperm count was determined by means of a Makler sperm counting chamber (Sefi‐Medical Instrument, Haifa, Israel). The resulting sperm count was expressed as ×10^6^/mL [35].

In the evaluation of sperm vitality, 10 μL of semen sample was placed on a slide and mixed with a drop of 1% Eosin Y. A total of 100 spermatozoa were counted on each slide and sperm vitality percentages were determined (light pink indicated live sperm, dark pink indicated non‐viable sperm). In the evaluation of sperm morphology, a total of 100 sperm cells per slide were examined in different areas at ×400 magnification, and abnormalities in the head, neck, and tail structures were determined [35, 36].

### 2.12 Statistical Analysis

The conformity of the data to a normal distribution was evaluated using the Shapiro–Wilk test, implemented in SPSS 23.0. The statistical analysis of data that were normally distributed was performed using ANOVA and Tukey's post hoc test. The results were presented as mean ± SEM, and a *p* value of less than 0.05 was considered statistically significant.

## 3 Results

### 3.1 Impacts of CHR on OS Levels and Nrf2 Signaling in Mice Exposed to ACP Intoxication

The data presented in Table 1 illustrate the impact of treatment protocols on testicular OS biomarkers in mice. The ACP treatment resulted in a notable elevation in LPO levels in comparison to the control group, accompanied by a discernible suppression of SOD, GSH, and GPx levels. The combination of low‐dose CHR treatment with ACP yielded a marked improvement in MDA and SOD levels, whereas the combination of high‐dose CHR treatment with ACP led to a substantial improvement in all OS biomarkers. Additionally, no adverse effects were discerned in the high‐dose CHR group in comparison to the control group.

**TABLE 1:** Effect of administration of CHR on markers of OS in testicular tissue.

	Control	CHR (20 mg/kg)	ACP	ACP + CHR (10 mg/kg)	ACP + CHR (20 mg/kg)
MDA (nmol/mg protein)	2.04 ± 0.24	2.38 ± 0.51	14.04 ± 1.65***	6.62 ± 0.61*^,###^	3.61 ± 1.17^###^
GSH (μg/mg protein)	14.48 ± 3.32	15.38 ± 0.89	4.95 ± 0.96*	7.27 ± 2.11	16.78 ± 1.66^##,+^
SOD (ng/mg protein)	0.51 ± 0.03	0.63 ± 0.09	0.19 ± 0.01**	0.43 ± 0.04^#^	0.52 ± 0.06^##^
GPx (IU/mg protein)	4.20 ± 0.44	4.93 ± 0.67	1.03 ± 0.22**	2.79 ± 0.30	3.87 ± 0.63^##^

*Note:* Data are represented as means ± SEM. Statistically significant from control group *p < 0.05, **p < 0.01, and ***p < 0.001. Statistically significant from ACP group ^#^ *p* < 0.05, ^##^ *p* < 0.01, and ^###^ *p* < 0.001. Statistically significant from ACP + CHR (10 mg/kg) group ^+^ *p* < 0.05.

As illustrated in Figure 1, the mice that were exposed to ACP exhibited a notable decline in testicular Nrf2, HO‐1, and GCLC levels in comparison to the control group. However, the administration of CHR in conjunction with ACP resulted in a notable improvement in the levels of Nrf2, HO‐1, and GCLC, which were observed to be lower in mice exposed to ACP alone.

**FIGURE 1:**
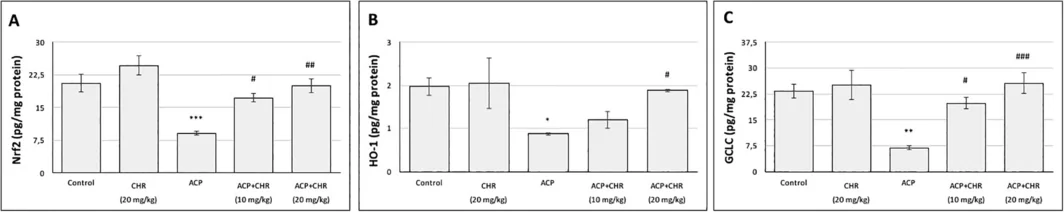
Effect of CHR and ACP administration on the levels of Nrf2 (A), HO‐1 (B) and GCLC (C) in the testicles of mice. Data are represented as means ± SEM (*n* = 6). Statistically significant from control group *p < 0.05, **p < 0.01, and ***p < 0.001. Statistically significant from ACP group #*p* < 0.05, ##*p* < 0.01, and ###*p* < 0.001.

### 3.2 Impacts of CHR on Inflammation Levels in Mice Exposed to ACP Intoxication

As illustrated in Figure 2, only ACP exposure resulted in a notable elevation in the levels of HMGB1, NF‐κB, IL‐6, and MPO in the testicular tissue of mice when compared to the levels observed in control mice. However, the administration of CHR in conjunction with ACP resulted in a notable reduction in inflammatory biomarker levels relative to mice exposed solely to ACP.

**FIGURE 2:**
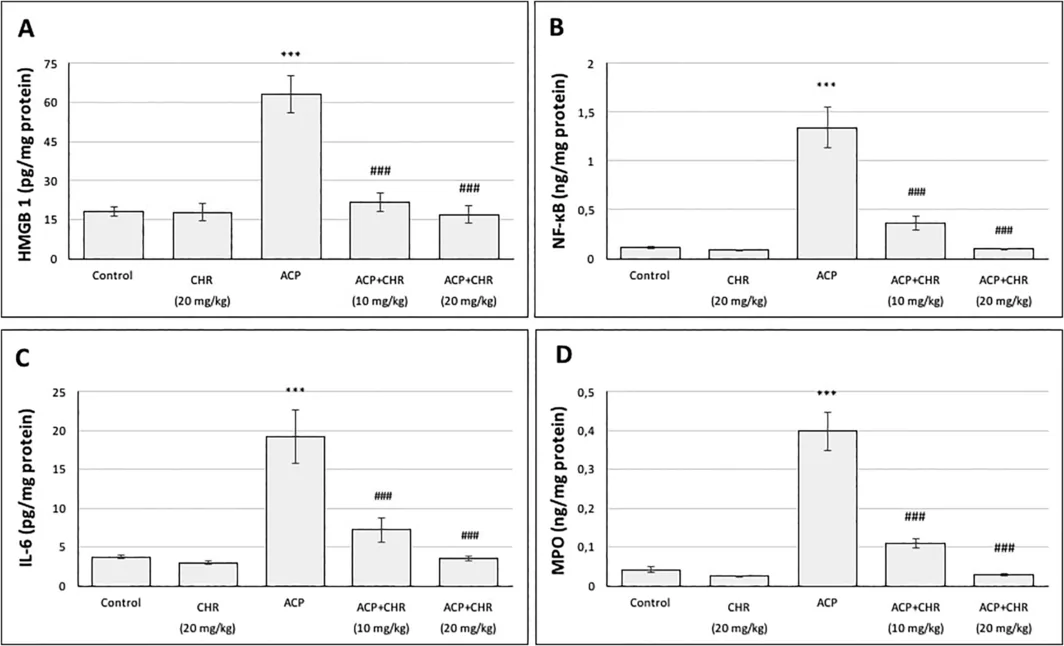
Effect of CHR and ACP administration on the levels of HMGB1 (A), NF‐κB (B), IL‐6 (C) and MPO (D) in the testicles of mice. Data are represented as means ± SEM (*n* = 6). Statistically significant from control group ***p < 0.001. Statistically significant from ACP group ###*p* < 0.001.

### 3.3 Impacts of CHR on ERS and Apoptosis Markers in Mice Exposed to ACP Intoxication

As illustrated in Figure 3, only ACP exposure resulted in a notable elevation in the levels of HSPA5, ATF6, DDIT3, and CASP3 in the testicular tissue of mice when compared to the levels observed in control mice. However, the administration of CHR in conjunction with ACP resulted in a notable reduction in ERS and apoptosis biomarker levels relative to mice exposed solely to ACP.

**FIGURE 3:**
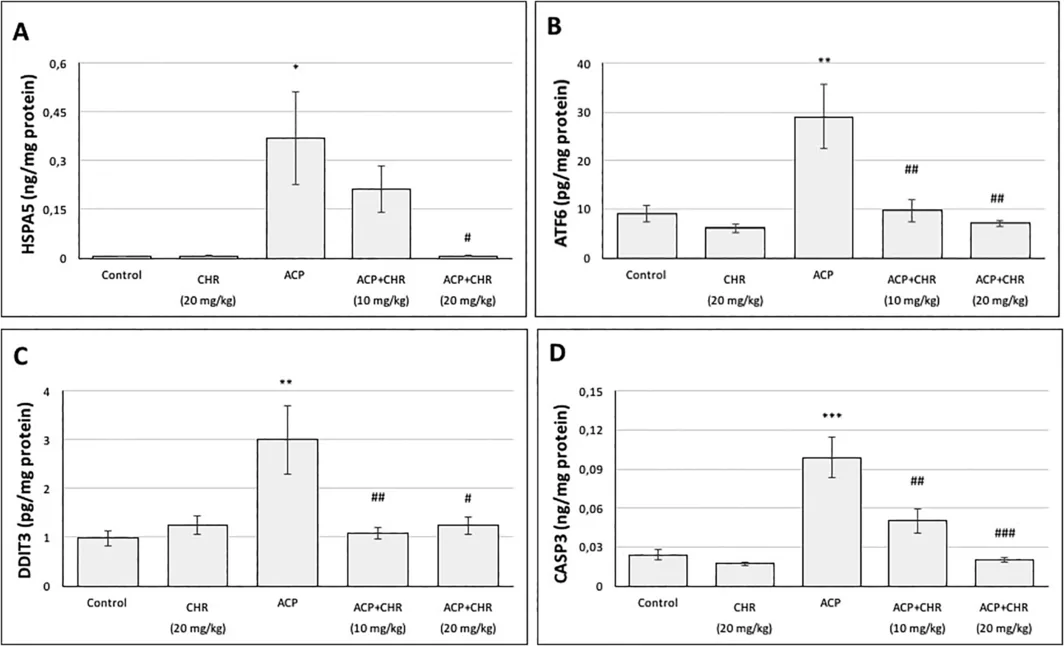
Effect of CHR and ACP administration on the levels of HSPA5 (A), ATF6 (B), DDIT3 (C) and CASP3 (D) in the testicles of mice. Data are represented as means ± SEM (*n* = 6). Statistically significant from control group *p < 0.05, **p < 0.01 and ***p < 0.001. Statistically significant from ACP group #*p* < 0.05, ##*p* < 0.01 and ###*p* < 0.001.

### 3.4 Impacts of CHR on Male Reproductive Hormones in Mice Exposed to ACP Intoxication

As illustrated in Table 2, the administration of ACP resulted in a notable decline in serum TES and INHB levels in mice, when compared to the control group. However, concurrent treatment with CHR (20 mg/kg) led to a significant restoration of serum reproductive hormone levels in comparison to the ACP‐treated group.

**TABLE 2:** Effect of administration of CHR on serum TES, and INHB levels.

	Control	CHR (20 mg/kg)	ACP	ACP + CHR (10 mg/kg)	ACP + CHR (20 mg/kg)
TES (ng/mL)	10.79 ± 0.96	8.50 ± 0.55	2.10 ± 0.80***	5.22 ± 0.17**	9.00 ± 0.66^###,+^
INHB (pg/mL)	1464.9 ± 72.7	1393.7 ± 44.1	430.6 ± 112.5***	754.3 ± 145.3***	1469.8 ± 23.3^###,+++^

*Note:* Data are represented as means ± SEM. Statistically significant from control group **p < 0.01 and ***p < 0.001. Statistically significant from ACP group ^###^ *p* < 0.001. Statistically significant from ACP + CHR (10 mg/kg) group ^+^ *p* < 0.05 and ^+++^ *p* < 0.001.

### 3.5 Impacts of CHR on Histological Lesions and Apoptosis in Mice Exposed to ACP Intoxication

The effects of ACP and CHR treatments on testicular tissue were determined by H&E and PAS staining methods, and the effects on apoptosis were determined by the TUNEL method. The results are presented in Figure 4 and Table 3. Despite the control and CHR (per se, 20 mg/kg) groups displaying normal seminiferous tubule and interstitial area morphology, rare areas of accumulation of seminiferous tubule epithelial cells towards the lumen were observed. Furthermore, the results of PAS staining demonstrated that the seminiferous tubule basement membrane exhibited prominent and regular characteristics in both the control and CHR (per se, 20 mg/kg) groups. In the H&E preparations of the ACP group, there was frequent observation of the following: accumulation of the seminiferous tubule epithelium towards the lumen, dissociation between the epithelial cells, and a decrease in the amount of spermatozoa in the lumen (as indicated by lower Johnsen scores). In the PAS staining preparations of the ACP group, the basement membrane could not be clearly observed in some places. In the H&E preparations of the ACP + CHR (10 mg/kg) group, dissociation and irregularities were observed between the seminiferous tubule epithelial cells. Conversely, PAS preparations of the aforementioned group exhibited a distinct basement membrane structure, analogous to that observed in the control group. In the H&E sections of the ACP + CHR (20 mg/kg) group, although testicular seminiferous tubule and interstitial area morphology close to normal was observed (as indicated by higher Johnsen scores), seminiferous tubule epithelial cells were occasionally piled up towards the lumen and small amounts of dissociation between seminiferous tubule epithelial cells were observed. Conversely, PAS preparations of this group exhibited a distinct basement membrane structure, analogous to that observed in the control group.

**FIGURE 4:**
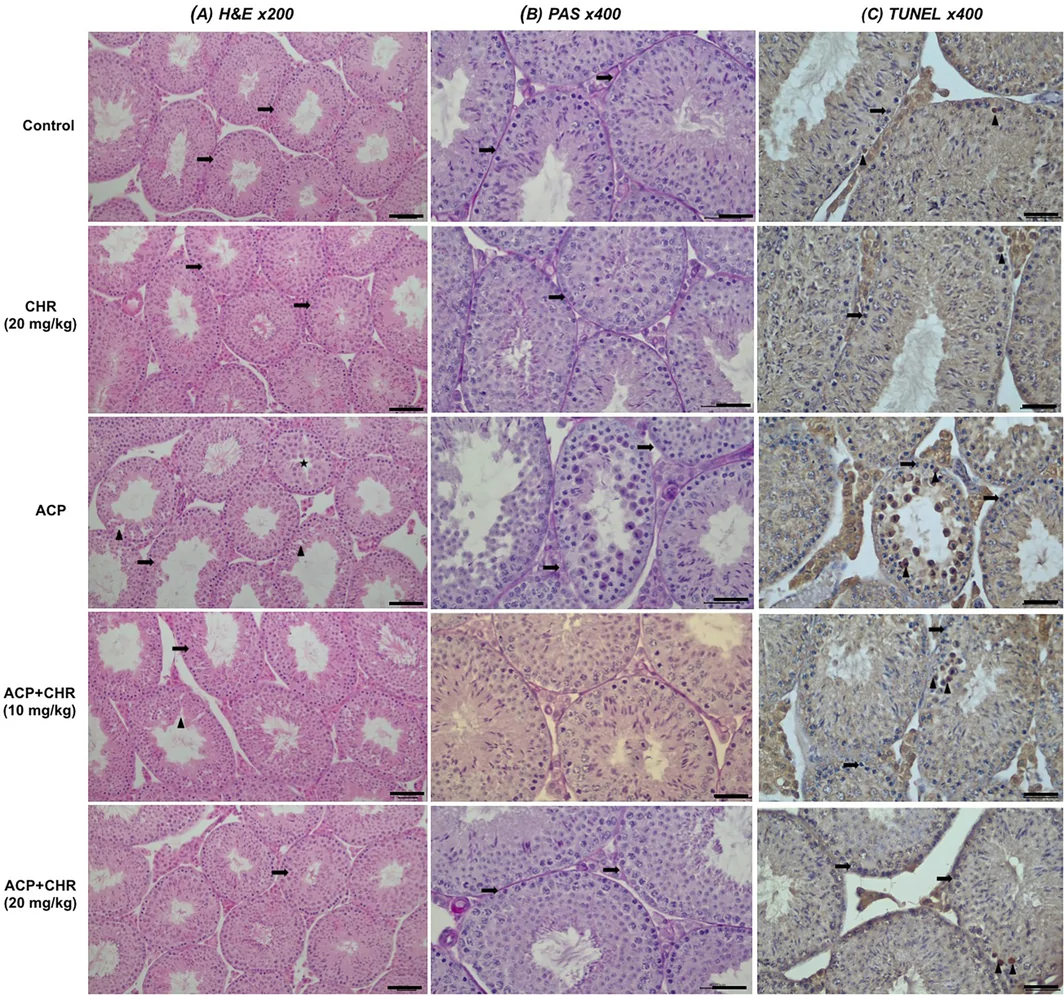
The light microscopy and TUNEL images of testicles from the control and experimental groups (*n* = 6). (A) H&E staining (×200); black arrow: Seminiferous tubule, black arrowhead: Dissociation between seminiferous tubule epithelial cells, star: accumulation towards the lumen in seminiferous tubule epithelium. (B) PAS staining (×400); black arrow: basement membrane of seminiferous tubule. (C) TUNEL staining (×400); black arrow: TUNEL (−) cell, black arrowhead: TUNEL (+) cell.

**TABLE 3:** Effect of administration of CHR on histopathological scores, AI, and sperm parameters.

	Control	CHR (20 mg/kg)	ACP	ACP + CHR (10 mg/kg)	ACP + CHR (20 mg/kg)
Frequency of germinal epithelial cells observed in the lumen	0.17 ± 0.17	0.17 ± 0.17	1.67 ± 0.21***	0.83 ± 0.17*^,##^	0.50 ± 0.22^###^
Accumulatio*n* of seminiferous tubule epithelium towards the lumen	0.33 ± 0.21	0.33 ± 0.21	2.00 ± 0.26***	1.17 ± 0.17**^,##^	0.83 ± 0.17^###^
Vasocongestion	0.17 ± 0.17	0.33 ± 0.21	1.00 ± 0.37*	0.50 ± 0.22	0.33 ± 0.21
Irregularity and dissociation between seminiferous tubule epithelial cells	0.33 ± 0.21	0.50 ± 0.22	2.17 ± 0.17***	1.33 ± 0.21**^,##^	0.83 ± 0.17^###^
Johnsen scores	9.67 ± 0.21	9.67 ± 0.21	6.83 ± 0.31***	8.33 ± 0.21**^,##^	9.33 ± 0.21^###,+^
AI	3.17 ± 0.31	3.33 ± 0.41	22.50 ± 0.96***	7.54 ± 0.56***^,###^	3.67 ± 0.21^###,+++^
Sperm count (×10^6^/mL)	48.17 ± 1.58	49.50 ± 2.45	25.17 ± 1.87***	35.67 ± 2.45**^,#^	42.17 ± 2.80^###^
Sperm vitality rate (%)	73.67% ± 1.61%	71.67% ± 1.12%	37.50% ± 2.43%***	43.50% ± 2.95%***	52.50% ± 2.59%***^,###^
Sperm morphology (% abnormal)	20.83% ± 1.01%	20.17% ± 0.75%	62.00% ± 5.99%***	48.17% ± 4.09%***^,#^	29.50% ± 1.36%^###,++^

*Note:* Data are represented as means ± SEM (*n* = 6). Statistically significant from control group **p < 0.01 and ***p < 0.001. Statistically significant from ACP group ^#^ *p* < 0.05, ^##^ *p* < 0.01, and ^###^ *p* < 0.001. Statistically significant from ACP + CHR (10 mg/kg) group ^+^ *p* < 0.05, ^++^ *p* < 0.01, and ^+++^ *p* < 0.001.

Abbreviations: ACP, acetamiprid; AI, apoptotic index, CHR: chrysin.

TUNEL analysis results of the control and CHR (per se, 20 mg/kg) groups revealed that a small number of apoptotic cells were detected in the seminiferous tubule epithelial cells. Conversely, TUNEL (+) apoptotic cells were frequently observed in the germinal epithelial cells in the ACP group (as indicated by the elevated AI). However, it was determined that CHR treatments (especially in the ACP + CHR [20 mg/kg] group) significantly reduced the number of ACP‐induced TUNEL (+) apoptotic cells (as indicated by the decreased AI).

### 3.6 Impacts of CHR on Sperm Count, Viability, and Morphology in Mice Exposed to ACP Intoxication

The effects of ACP and CHR applications on sperm parameters are presented in Table 3. Compared with the control group, administration of CHR (20 mg/kg) alone did not result in a negative effect on sperm count, viability, and morphology. However, administration of ACP significantly reduced sperm count and viability, and increased the number of abnormal sperm. However, concurrent CHR treatments with ACP (with the 20 mg/kg dose being more effective) greatly improved the pathological sperm parameter values caused by ACP.

## 4 Discussion

The findings of the present study substantiated the hypothesis that ACP induces testicular damage by suppressing Nrf2 and elevating the levels of OS, inflammation, ERS, and apoptosis. Moreover, administration of ACP resulted in a marked decline in serum reproductive hormone levels. However, CHR treatments demonstrated that the restoration of testicular tissue, sperm parameters, and serum reproductive hormone levels was significantly achievable by the elimination of Nrf2 suppression.

The present study showed that ACP exposure led to a significant reduction in serum TES and INHB levels, caused significant changes to the structure of the testicles, increased the number of TUNEL positive cells, and had a negative effect on sperm parameters. These observations align with those reported in previous studies [21, 37, 38]. TES is a hormone that plays a crucial role in male reproductive function [39]. INHB, on the other hand, is regarded as a significant marker for the functional status of seminiferous tubules [21]. Cyclic adenosine monophosphate (cAMP) is a molecule that regulates TES biosynthesis by modulating enzymes involved in the steroidogenesis process. There is a relationship between increased levels of ROS and depletion of cAMP [3, 38]. The reduction in serum hormone levels observed in the ACP group may be attributable to the heightened OS findings documented in this group. However, CHR treatments (especially at a dose of 20 mg/kg) led to a big improvement in hormone levels in the blood, sperm quality, and a reduction in testicular damage and the number of TUNEL‐positive cells compared to those seen in mice treated with ACP. The elevated levels of reproductive hormones observed in the CHR treatment groups relative to the ACP group may be attributed to the reduction in OS levels in these groups. As similar to our findings, previous studies have also demonstrated that CHR exerts a protective effect on the testicles [17, 18, 19, 31].

It has been postulated that OS is the primary mechanism responsible for ACP‐induced tissue injury [1, 21]. ACP has been demonstrated to induce oxidative damage, including LPO, by increasing ROS levels, particularly superoxide, and by suppressing the antioxidant system [3]. Furthermore, evidence indicates that ACP enhances the susceptibility of tissue to oxidative damage by reducing the expression of Nrf2 and its target proteins [8, 14]. In accordance with the aforementioned findings, the results demonstrated a reduction in Nrf2 and antioxidant system activity, accompanied by an increase in LPO levels within the ACP group. However, the concomitant administration of ACP and CHR resulted in a reduction in LPO levels, which was attributed to the modulation of Nrf2 and antioxidant system capacity. This may be attributed to the direct free radical scavenging activity of the hydroxyl groups present in the CHR structure [15, 16]. Furthermore, the improvement in LPO levels may be attributed to the activation of the antioxidant system resulting from the modulation of Nrf2 signaling by CHR. The current findings are in accordance with those of other studies, which have demonstrated that CHR exerts a protective effect through the modulation of Nrf2 [40, 41].

It has been demonstrated that inflammation is a significant factor in the toxicity of ACP [3, 6]. HMGB1 is a non‐histone chromosomal protein that plays a role in the processes of DNA repair and transcription. In addition to its nuclear functions, the release of it from damaged cells has been observed to result in the activation of NF‐κB, which in turn has been linked to an increase in proinflammatory cytokines, including IL‐6 [42]. Thefore, the inhibition of the HMGB1/NF‐κB/IL‐6 signaling pathway represents a pivotal strategy for the prevention of chronic inflammation‐induced tissue damage [43]. In accordance with previous reports [6, 7, 8], ACP exposure resulted in the activation of the inflammatory cascade, as evidenced by elevated levels of HMGB1, NF‐κB, IL‐6, and MPO in mice. Nevertheless, CHR treatments were found to have a marked inhibitory effect on inflammatory markers. It is possible that the in vivo antioxidant activity contributed to the anti‐inflammatory effect of CHR [15, 16]. The reduction in OS levels observed in the CHR groups provides support for the validity of this hypothesis. Secondly, given that Nrf2 provides negative regulation of NF‐κB [44], the observed decrease in inflammation in the CHR groups may be attributed to the modulation of Nrf2 by CHR in these groups. Our findings were also consistent with those of previous studies, which demonstrated the anti‐inflammatory activity of CHR in various in vivo models [17, 22].

The ER is a cellular organelle that plays a crucial role in protein synthesis, folding, and maturation processes [9]. The disruption of ER homeostasis, which may result from factors such as infection, OS, calcium imbalance, and inflammation, is referred to as ERS [10]. In cells, the detection of ERS is facilitated by a number of sensors, including ATF6, which then initiates the unfolding protein response (UPR) [9]. In physiological conditions, ATF6 is maintained in an inactive state through its interaction with HSPA5 [30]. However, when ERS occurs, ATF6 is released from this state. Following its activation in the Golgi apparatus, it is transported to the nucleus, where it activates genes that will restore ER homeostasis [9]. In the event of prolonged and irreparable ERS, DDIT3 expression is increased by ATF6, leading to the induction of apoptosis [10]. Recent studies have indicated an increase in ERS and apoptosis in response to ACP toxicity [4, 11]. In line with this approach, our findings demonstrated an elevation in ERS and apoptosis levels in mice subjected to ACP treatment. Nevertheless, CHR treatments were observed to inhibit the levels of ERS and apoptosis. The ERS and apoptosis modulatory activity of CHR can be primarily attributed to the antioxidant and anti‐inflammatory properties of CHR [15, 16]. Secondly, it is established that Nrf2 plays a role in alleviating cellular ERS by up‐regulating components of proteasome‐mediated ER‐associated degradation [10]. The observation of elevated Nrf2 levels in the treatment groups in which ACP and CHR were administered in conjunction, in comparison to the ACP group, may lend support to the hypothesis that this potential pathway may be involved in the reduction of ERS and apoptosis. Furthermore, these findings were in accordance with previous reports indicating that CHR may alleviate ERS and apoptosis in vivo [31, 45, 46].

## 5 Conclusion

CHR may exert a protective effect against ACP‐induced testicular toxicity through Nrf2 modulation (Figure 5). However, further comprehensive experimental and clinical studies are necessary to substantiate this hypothesis and to ascertain its potential as a protective molecule in preventing testicular damage.

**FIGURE 5:**
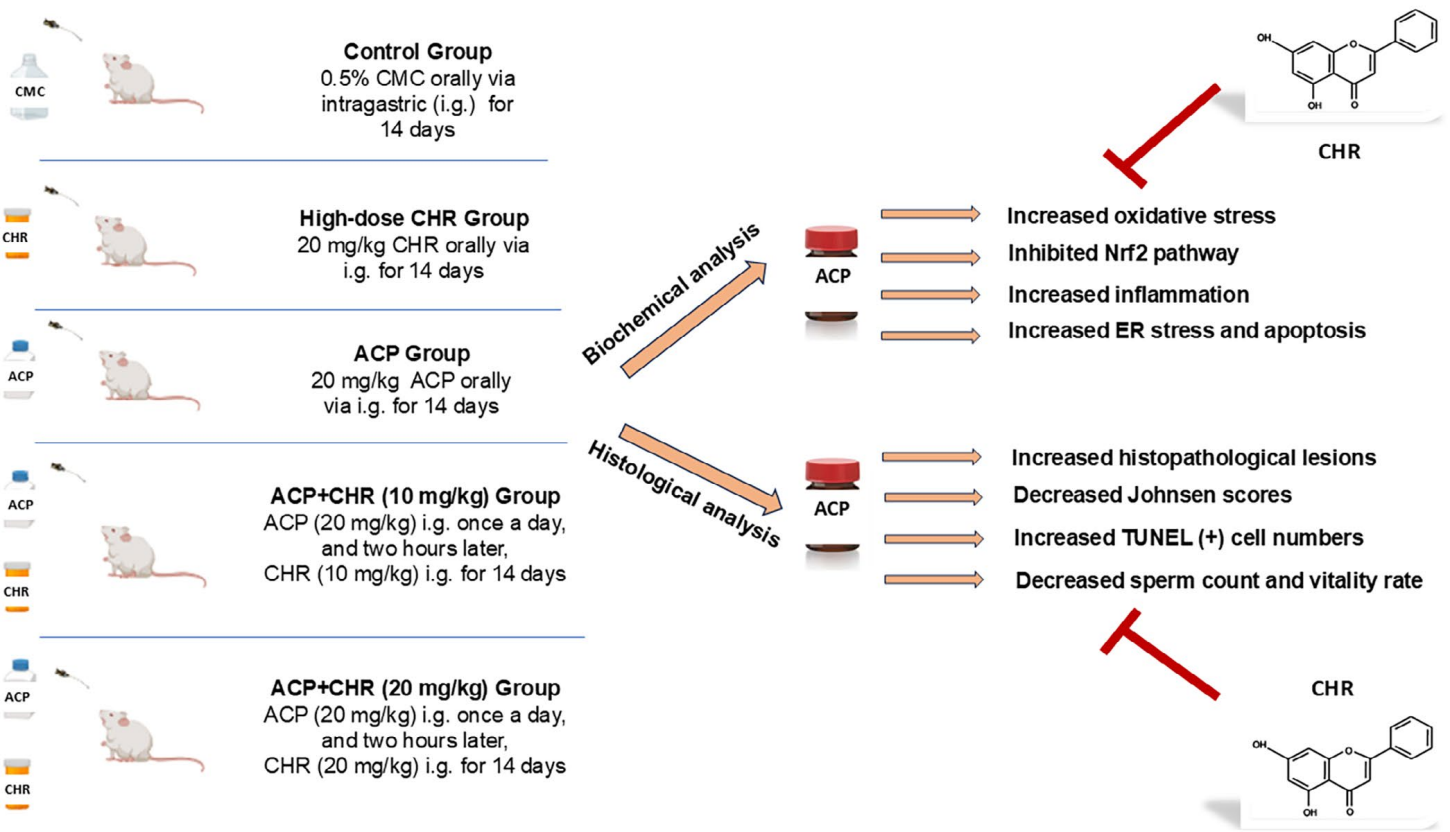
A visual summary of the experimental design and key findings of the study (ACP: Acetamiprid, CHR: Chrysin, CMC: Carboxymethylcellulose).

## Author Contributions

**Selim Demir:** conceptualization, project administration, investigation, methodology, supervision, formal analysis, writing – original draft. **Nihal Turkmen Alemdar:** investigation, methodology, formal analysis. **Esin Yulug:** methodology, investigation, formal analysis, writing – review and editing. **Ali Kulaber:** methodology, investigation. **Elif Ayazoglu Demir:** methodology, investigation. **Nadire Sevdenur Erdogan:** methodology, investigation. **Ahmet Mentese:** software, methodology, formal analysis, supervision. **Yuksel Aliyazicioglu:** methodology, writing – review and editing. All authors gave final approval of the version to be published.

## Funding

This study was supported by the Office of Scientific Research Projects of Karadeniz Technical University (project number: TSA‐2024‐11112).

## Ethics Statement

This study was approved by the Local Animal Research Ethics Committee of Karadeniz Technical University (protocol no: 2024/17) and conducted in accordance with the animal research reporting of in vivo experiments (ARRIVE) guidelines and the UK Animals (Scientific Procedures) Act 1986 and related guidelines, EU Directive 2010/63/EU for animal experimentation and National Institutes of Health guidelines.

## Conflicts of Interest

The authors declare no conflicts of interest.

## Data Availability

The data that support the findings of this study are available from the corresponding author upon reasonable request.
